# Impact of Rifaximin, a Gut-Selective Antibiotic, on Gastric Intestinal Metaplasia: A Retrospective Study

**DOI:** 10.3390/jcm15135282

**Published:** 2026-07-06

**Authors:** Gokhan Aydin, Bengisu Ulu Karasu, Selcuk Takir, Ahmet Cumhur Dulger

**Affiliations:** 1 Department of Internal Medicine, Division of Gastroenterology, Faculty of Medicine, Giresun University, Giresun 28200, Turkey; bengisulu@gmail.com (B.U.K.); acdulger@gmail.com (A.C.D.); 2 Department of Pharmacology, Faculty of Medicine, Giresun University, Giresun 28200, Turkey; selcuk.takir@giresun.edu.tr

**Keywords:** rifaximin, gastric intestinal metaplasia, gut microbiota, *Helicobacter pylori*, premalignant gastric lesions, gastric carcinogenesis

## Abstract

**Background/Aim:** Rifaximin is a gut-selective antibiotic with minimal systemic absorption that exerts its effects primarily within the gastrointestinal tract. Beyond its antimicrobial activity, rifaximin has been shown to possess anti-inflammatory and immunomodulatory properties. Gastric intestinal metaplasia (GIM) is a premalignant lesion associated with chronic gastric inflammation and *Helicobacter pylori* infection and is considered an important step in gastric carcinogenesis. Given the regulatory effects of rifaximin on the gut microbiota and mucosal inflammation, its potential impact on gastric histopathological alterations remains poorly understood. This study aimed to evaluate the effects of rifaximin therapy on gastric histopathological findings, particularly gastric intestinal metaplasia. **Methods:** In this retrospective single-center study, patients who received rifaximin therapy for various gastrointestinal indications and underwent upper gastrointestinal endoscopy with gastric biopsies both before and after treatment were included. Demographic characteristics, rifaximin treatment data, and histopathological findings were obtained from hospital records. Pre- and post-treatment biopsy findings were compared with respect to gastric intestinal metaplasia, dysplasia, and *Helicobacter pylori* positivity. **Results:** A total of 80 patients (mean age: 62.4 ± 11.9 years; 58.8% male) were included. Following rifaximin therapy, gastric intestinal metaplasia demonstrated significant regression (*p* < 0.001). Complete regression was observed in 45 patients (56.2%), partial regression in 12 patients (15.0%), stable disease in 14 patients (17.5%), and progression in 9 patients (11.3%). *Helicobacter pylori* positivity decreased from 42.5% to 31.2% (*p* = 0.041), whereas no significant change was observed in dysplasia rates (*p* = 0.564). No significant correlation was found between biopsy interval and the degree of intestinal metaplasia regression (Spearman’s r = −0.057, *p* = 0.617). **Conclusions:** Rifaximin therapy was associated with regression in both the presence and severity of gastric intestinal metaplasia. These findings provide preliminary evidence supporting further investigation of rifaximin in gastric intestinal metaplasia. However, the results should be considered hypothesis-generating, and larger prospective controlled studies are required to establish a causal relationship and further elucidate the underlying mechanisms.

## 1. Introduction

Gastric intestinal metaplasia (GIM) is a premalignant lesion characterized by the replacement of normal gastric epithelium with intestinal-type epithelium and is considered a key step in the development of gastric adenocarcinoma. The development of GIM is closely associated with chronic gastric inflammation, particularly Helicobacter pylori infection, and represents part of the multistep Correa cascade progressing through atrophy, metaplasia, dysplasia, and carcinoma [1,2].

Although *Helicobacter pylori* eradication has been shown to reduce gastric inflammation, decrease the risk of gastric cancer, and potentially slow disease progression, no standard pharmacological therapy has yet been proven effective in promoting regression of intestinal metaplasia. Furthermore, intestinal metaplasia has been proposed to represent an “irreversible stage” in the process of gastric carcinogenesis in at least a subset of patients [3,4].

In recent years, increasing attention has been directed toward the role of the gut microbiota and mucosal immune responses in the pathogenesis of gastrointestinal diseases. Alterations in microbial composition and chronic low-grade inflammation are thought to contribute not only to disorders of the lower gastrointestinal tract but also to pathologies affecting the upper gastrointestinal tract [5,6,7]. In this context, therapeutic approaches aimed at reducing microbial burden and modulating mucosal inflammation have emerged as potential strategies capable of influencing the course of gastric premalignant lesions.

Rifaximin is a minimally absorbed, broad-spectrum antibiotic that acts locally within the intestinal lumen and is widely used in clinical practice for the treatment of hepatic encephalopathy, irritable bowel syndrome, and small intestinal bacterial overgrowth [8,9]. In addition to its antimicrobial activity, rifaximin has been reported to exert anti-inflammatory and immunomodulatory effects within the intestinal mucosa, particularly through activation of the pregnane X receptor (PXR), resulting in suppression of NF-κB signaling and reduced expression of pro-inflammatory cytokines [10,11,12]. Rifaximin has also been shown to enhance epithelial barrier function and reduce inflammation-associated mucosal injury [13,14].

Although the effects of rifaximin on the gut microbiota and intestinal inflammation have been well characterized [10,13], its potential impact on the gastric mucosa, particularly on histopathological alterations such as gastric intestinal metaplasia, remains poorly understood. Given the established role of chronic inflammation and microbial factors in the pathogenesis of GIM, it is conceivable that the regulatory effects of rifaximin on these processes may indirectly influence the gastric mucosa as well.

In light of these considerations, the present study aimed to retrospectively evaluate the potential effects of rifaximin therapy on histopathological changes in the gastric mucosa, particularly gastric intestinal metaplasia, by comparing endoscopic biopsy findings obtained before and after treatment in patients receiving rifaximin for various clinical indications.

## 2. Materials and Methods

### 2.1. Study Design, Population, and Setting

This study was designed as a single-center, retrospective, descriptive investigation conducted at Giresun Training and Research Hospital. The study was based on the review of hospital information system records and archived medical data.

A total of 80 patients (mean age: 62.4 ± 11.9 years; 58.8% male) with histopathologically confirmed gastric intestinal metaplasia (GIM) at baseline who subsequently received rifaximin therapy for small intestinal bacterial overgrowth (SIBO), irritable bowel syndrome (IBS), chronic diarrhea, or similar gastrointestinal indications were included. All patients underwent upper gastrointestinal endoscopy with gastric biopsy sampling both before and after rifaximin therapy. Repeat upper gastrointestinal endoscopy with gastric biopsy was performed according to routine clinical practice, based on the treating physician’s clinical judgment and routine clinical indications documented in the medical records. Changes in gastric intestinal metaplasia observed on follow-up endoscopies performed at least six months after baseline were retrospectively evaluated.

### 2.2. Patient Selection

Patients who received rifaximin therapy for various gastrointestinal indications within the previous five years were identified through the hospital information system and retrospectively screened for eligibility.

**Figure 1:**
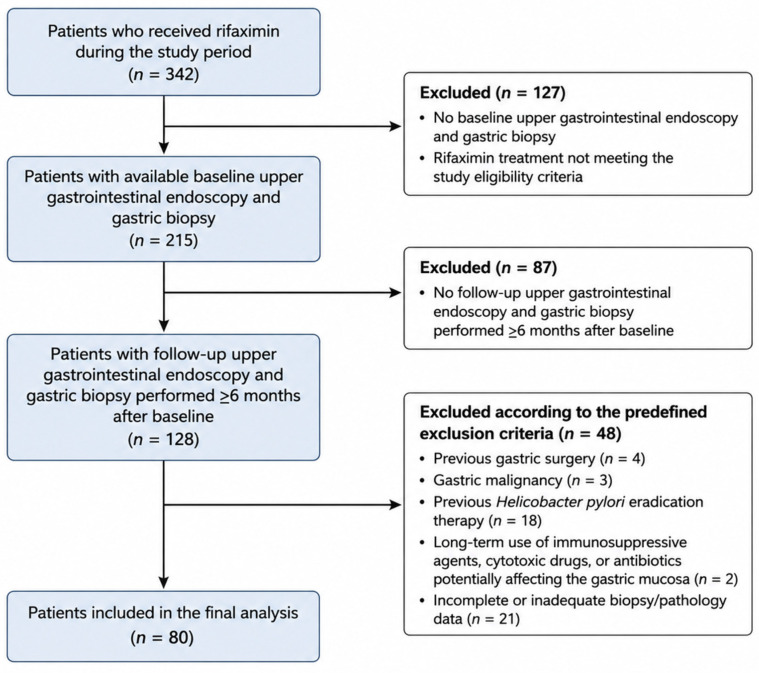
Flow diagram of patient selection. The diagram illustrates the screening process, application of the predefined inclusion and exclusion criteria, and selection of the final study population.

A total of 342 patients were screened. After application of the predefined inclusion and exclusion criteria, 80 patients were included in the final analysis (Figure 1).

### 2.3. Inclusion Criteria

- Treatment with rifaximin for SIBO, IBS, chronic diarrhea, or similar gastrointestinal indications.
- Rifaximin administration for at least three treatment courses, each consisting of 15 days at a dose of 200 mg three times daily (minimum cumulative treatment duration of 45 days and minimum daily dose of 600 mg).
- Availability of upper gastrointestinal endoscopy and gastric biopsy findings both before and after rifaximin therapy.
- Histopathological diagnosis of gastric intestinal metaplasia at baseline endoscopy.
- Follow-up endoscopy performed at least six months after the baseline examination.
- No *Helicobacter pylori* eradication therapy administered between the two endoscopic evaluations.
- Availability of pathology reports suitable for histopathological assessment.
- Age ≥ 18 years.

### 2.4. Exclusion Criteria

- History of gastric malignancy.
- Previous gastric surgery.
- Receipt of *Helicobacter pylori* eradication therapy during the rifaximin treatment period.
- Long-term use of immunosuppressive agents, cytotoxic drugs, or antibiotics potentially affecting the gastric mucosa.
- Incomplete or inadequate biopsy/pathology data.
- Pregnancy or breastfeeding.
- Age < 18 years.

### 2.5. Data Collection and Histopathological Evaluation

Demographic characteristics (age and sex), clinical variables (duration and indication of rifaximin therapy), and endoscopy dates were obtained from the hospital information system and electronic medical records. Because of the retrospective study design, detailed information regarding treatment adherence, retreatment patterns, and individual cumulative rifaximin exposure beyond the predefined inclusion criteria was not consistently available for all patients.

Gastric biopsy findings obtained before and after rifaximin therapy were reviewed and compared. For the purposes of this study, gastric intestinal metaplasia was considered present if detected in any biopsy specimen obtained from the gastric antrum and/or corpus. Follow-up biopsies were obtained from the same anatomical gastric region (antrum or corpus) as the baseline biopsy whenever repeat endoscopy was performed. When multiple biopsy specimens from the same examination were available, the highest reported grade of intestinal metaplasia was used for histopathological classification. Thus, paired comparisons were based on the highest grade identified within the corresponding anatomical region before and after rifaximin therapy.

The following histopathological parameters were evaluated:

- Severity of gastric intestinal metaplasia, classified according to existing pathology reports as mild (+), moderate (++), or severe (+++).
- Presence of dysplasia.
- *Helicobacter pylori* positivity.

*Helicobacter pylori* positivity was determined based on routine histopathological examination of the gastric biopsy specimens obtained from the antrum and/or corpus during the same endoscopic procedures used to evaluate gastric intestinal metaplasia and dysplasia.

Histopathological assessments were based on routine pathology reports generated during standard clinical practice. The pathological evaluations were performed by board-certified pathologists according to the routine diagnostic standards of the pathology department. Standardized pathological diagnostic criteria were routinely applied for the assessment of gastric intestinal metaplasia and related histopathological findings.

No additional biopsies or interventions were performed, and all data were obtained from existing medical records. Patients were included regardless of baseline *Helicobacter pylori* status, provided that they had not received *H. pylori* eradication therapy during the follow-up period and had been treated exclusively with rifaximin.

### 2.6. Statistical Analysis

Statistical analyses were performed using IBM SPSS Statistics for Windows, Version 26.0 (IBM Corp., Armonk, NY, USA). Normality of continuous variables was assessed using the Kolmogorov–Smirnov test and skewness–kurtosis values. Normally distributed variables were presented as mean ± standard deviation, whereas non-normally distributed variables were expressed as median (minimum–maximum).

For comparisons between pre- and post-treatment findings, paired-samples t-tests were used for normally distributed continuous variables, Wilcoxon signed-rank tests for non-normally distributed continuous variables, and McNemar or Fisher’s exact tests for categorical variables, as appropriate.

Associations between biopsy interval and histopathological changes were evaluated using Spearman correlation analysis.

A two-sided *p*-value < 0.05 was considered statistically significant.

## 3. Results

The baseline demographic and clinical characteristics of the study population, including the primary clinical indications for rifaximin therapy, are summarized in Table 1.

When gastric histopathological findings before and after rifaximin therapy were compared, a marked improvement in intestinal metaplasia was observed. While intestinal metaplasia was present in all patients at baseline, complete regression was documented in 56.2% of patients following treatment. In addition, the proportion of patients with mild (+) intestinal metaplasia decreased from 55.0% to 16.2%, moderate (++) intestinal metaplasia from 27.5% to 20.0%, and severe (+++) intestinal metaplasia from 17.5% to 7.5%. These changes were statistically significant (*p* < 0.001) (Table 2).

With respect to dysplasia, the prevalence decreased from 2.5% before treatment to 1.2% after treatment. However, dysplasia rates were low in both periods, and no statistically significant difference was observed (*p* = 0.564) (Table 2).

The prevalence of *Helicobacter pylori* positivity decreased from 42.5% before treatment to 31.2% after treatment, and this change was statistically significant (*p* = 0.041). Nevertheless, the magnitude of this reduction appeared less pronounced than the improvement observed in intestinal metaplasia (Table 2).

Gastric intestinal metaplasia response patterns were evaluated after rifaximin therapy. Complete regression was observed in 56.2% of patients, partial regression in 15.0%, stable disease in 17.5%, and progression in 11.3% (Table 3). No significant correlation was observed between the interval between biopsies and the degree of intestinal metaplasia regression (Spearman’s r = −0.057, *p* = 0.617), suggesting that differences in follow-up duration were unlikely to have substantially influenced the observed histopathological outcomes.

Patient-level transitions between baseline and follow-up gastric intestinal metaplasia grades are presented in Table 4. Regression was observed across all baseline severity categories, although complete regression was more frequent among patients with mild baseline intestinal metaplasia than among those with moderate or severe disease.

## 4. Discussion

In this retrospective study, the effects of rifaximin therapy on histopathological findings of the gastric mucosa, particularly gastric intestinal metaplasia (GIM), were evaluated. The findings suggest that rifaximin use may be associated with regression in both the presence and severity of intestinal metaplasia. In our cohort, intestinal metaplasia, which was present in all patients at baseline, completely disappeared in a substantial proportion of patients following treatment, while the remaining patients demonstrated a reduction in metaplasia severity. These findings suggest a possible association between rifaximin therapy and favorable histopathological changes in gastric premalignant mucosal alterations. Notably, complete regression of intestinal metaplasia was observed in 56.2% of patients following rifaximin therapy. Patient-level transition analysis showed that regression occurred across all baseline severity categories, although complete regression was more frequently observed among patients with mild baseline intestinal metaplasia than among those with moderate or severe disease (Table 4). Although direct comparisons are difficult because of differences in study populations, follow-up duration, and methodology, this finding appears noteworthy given the limited and inconsistent evidence regarding regression of gastric intestinal metaplasia reported in previous studies [3,15].

Gastric intestinal metaplasia is an important premalignant histopathological alteration that develops as a consequence of chronic inflammation and is associated with an increased risk of gastric cancer. *Helicobacter pylori* infection and the persistent inflammatory response it induces are considered key driving factors in this process. However, effective pharmacological strategies capable of promoting regression of intestinal metaplasia remain limited. Given the potential contribution of microbial and inflammatory factors to the pathogenesis of gastric intestinal metaplasia, agents that modulate the intestinal mucosa and gut microbiota may exert indirect effects on this process [3,15,16,17]. Therefore, investigating the potential impact of therapies that regulate inflammation and reduce microbial burden may provide valuable insights into novel approaches for the management of gastric intestinal metaplasia.

Rifaximin is a minimally absorbed, gut-selective antibiotic that exerts its effects primarily within the intestinal lumen. Beyond its antimicrobial activity, rifaximin has been shown to possess anti-inflammatory and immunomodulatory properties. Experimental and clinical studies have demonstrated that rifaximin activates the pregnane X receptor (PXR), leading to inhibition of NF-κB-mediated inflammatory signaling, reduction in pro-inflammatory cytokine expression, and enhancement of mucosal barrier function [10,13]. Considering these mechanisms, it is plausible that rifaximin may indirectly influence the course of gastric intestinal metaplasia by attenuating chronic inflammatory processes. Recent evidence further supports the concept that modulation of the gut microbiome may influence immune regulation, inflammatory signaling, and mucosal homeostasis across a variety of gastrointestinal and systemic disorders, providing additional biological plausibility for microbiota-targeted therapeutic approaches. Although direct evidence in gastric intestinal metaplasia remains limited, these findings support the hypothesis that gut microbiota modulation may indirectly influence gastric mucosal homeostasis [18].

Although the primary effects of rifaximin are exerted at the level of the small intestine and colon through modulation of microbial composition and suppression of mucosal inflammation, the interconnected nature of mucosal immune responses throughout the gastrointestinal tract suggests that these effects may also extend indirectly to the gastric mucosa. This hypothesis may be particularly relevant in gastric intestinal metaplasia, a condition characterized by the acquisition of intestinal-type epithelial features within the gastric mucosa.

In our study, a modest reduction in *Helicobacter pylori* positivity was also observed; however, this change appeared less pronounced than the regression observed in intestinal metaplasia. This observation raises the possibility that the potential effects of rifaximin on gastric intestinal metaplasia may be mediated primarily through indirect mechanisms, such as modulation of the gut microbiota and suppression of chronic inflammation, rather than through a direct antibacterial effect against *Helicobacter pylori*. The observed reduction in *H. pylori* positivity despite exclusion of eradication therapy may alternatively reflect sampling variability inherent to histopathological detection or false-negative histopathological findings, and therefore cannot be definitively attributed to a direct antimicrobial effect of rifaximin within the limitations of the present retrospective study. Furthermore, because of the retrospective study design and the relatively limited number of patients with changes in *H. pylori* status, additional stratified or sensitivity analyses were not considered sufficiently robust and should be addressed in future prospective studies.

No significant changes in dysplasia were observed between the pre-treatment and post-treatment periods. Nevertheless, the low prevalence of dysplasia at both time points and the absence of histopathological progression suggest that rifaximin therapy did not exert an adverse effect on premalignant progression during the study period.

In conclusion, the findings of this study suggest that rifaximin use may be associated with regression in both the presence and severity of gastric intestinal metaplasia. Given that gastric intestinal metaplasia is a premalignant condition characterized by the acquisition of intestinal-type epithelial features within the gastric mucosa, it is plausible that the regulatory effects of rifaximin on the gut microbiota and mucosal inflammation may indirectly influence gastric mucosal homeostasis.

Although a causal relationship cannot be established based on the present findings, the observed histopathological improvements provide preliminary evidence that supports further investigation of rifaximin in well-designed prospective, controlled, and mechanistic studies.

## 5. Limitations

This study has several important limitations. First, its retrospective single-center design may limit the generalizability of the findings. Second, because the study population consisted exclusively of patients who received rifaximin and underwent endoscopic follow-up, selection bias cannot be excluded, and the absence of an untreated control group precludes definitive conclusions regarding causality. Third, histopathological assessments were based on routine clinical pathology reports rather than centralized blinded re-review of paired biopsy specimens. Although follow-up biopsies were obtained from the same anatomical gastric region whenever repeat endoscopy was performed, sampling variability inherent to gastric intestinal metaplasia cannot be completely excluded. Finally, the interval between biopsies, duration of rifaximin therapy, and other potential confounding factors could not be fully standardized. In particular, information regarding concomitant use of proton pump inhibitors and other medications was not consistently available because of the retrospective study design and may represent an additional source of confounding.

In addition, although spontaneous regression of gastric intestinal metaplasia cannot be completely excluded, it is generally considered uncommon in the literature, and the magnitude of the observed regression warrants further investigation in well-designed prospective controlled studies.

Despite these limitations, the observed improvement demonstrated a consistent pattern across paired histopathological evaluations. However, the biological mechanisms that may underlie these observations remain speculative and require confirmation in prospective mechanistic studies. Nevertheless, the present results should be considered hypothesis-generating and interpreted with appropriate caution until confirmed by prospective controlled studies incorporating standardized biopsy protocols and centralized histopathological review.

To the best of our knowledge, this is among the first clinical studies to evaluate the potential association between rifaximin therapy and histopathological changes in gastric intestinal metaplasia in humans. The observed favorable histopathological findings provide preliminary clinical evidence that may serve as a foundation for future prospective controlled studies investigating the potential role of rifaximin in patients with gastric intestinal metaplasia.

## 6. Conclusions

Rifaximin therapy was associated with regression in both the presence and severity of gastric intestinal metaplasia. These findings should be considered hypothesis-generating and support further investigation of rifaximin in well-designed prospective, controlled, and mechanistic studies. Larger prospective controlled studies are required to establish whether a causal relationship exists and to further elucidate the underlying mechanisms involved.

## Figures and Tables

**Table 1:** Baseline demographic and clinical characteristics of the study population.

Variables	Total Cohort (*n* = 80)
Age, years, mean ± SD	62.4 ± 11.9
Male sex, *n* (%)	47 (58.8)
Female sex, *n* (%)	33 (41.2)
Interval between biopsies, months, mean ± SD	10.2 ± 7.3
**Indication for rifaximin therapy, *n* (%)**	
IBS/functional bowel disorder	30 (37.5)
Chronic diarrhea	20 (25.0)
Suspected or confirmed SIBO	19 (23.8)
Other gastrointestinal indications	11 (13.7)

Some patients had overlapping gastrointestinal symptoms; therefore, classification was based on the primary clinical indication documented in the medical records.

**Table 2:** Comparison of histopathological findings before and after rifaximin therapy.

Histopathological Findings	Before Rifaximin *n* (%)	After Rifaximin *n* (%)	*p*-Value
**Intestinal Metaplasia**			<0.001
Negative (−)	0 (0)	45 (56.2)	
Mild (+)	44 (55.0)	13 (16.2)	
Moderate (++)	22 (27.5)	16 (20.0)	
Severe (+++)	14 (17.5)	6 (7.5)	
**Dysplasia**			0.564
Negative (−)	78 (97.5)	79 (98.8)	
Positive (+)	2 (2.5)	1 (1.2)	
***Helicobacter pylori***			0.041
Negative (−)	46 (57.5)	55 (68.8)	
Positive (+)	34 (42.5)	25 (31.2)	

Abbreviations: (−), negative; (+), mild; (++), moderate; (+++), severe for intestinal metaplasia. For dysplasia and Helicobacter pylori, (−) indicates negative and (+) indicates positive findings.

**Table 3:** Response pattern of gastric intestinal metaplasia after rifaximin therapy.

Outcome After Rifaximin Therapy	*n* (%)
Complete regression	45 (56.2)
Partial regression	12 (15.0)
Stable disease	14 (17.5)
Progression	9 (11.3)

**Table 4:** Patient-level transition matrix of gastric intestinal metaplasia severity before and after rifaximin therapy.

Baseline	After: Negative (−)	After: Mild (+)	After: Moderate (++)	After: Severe (+++)	Total
**Mild (+)**	31 (70.5%)	6 (13.6%)	6 (13.6%)	1 (2.3%)	44
**Moderate (++)**	11 (50.0%)	4 (18.2%)	5 (22.7%)	2 (9.1%)	22
**Severe (+++)**	3 (21.4%)	3 (21.4%)	5 (35.7%)	3 (21.4%)	14

Abbreviations: (−), negative; (+), mild; (++), moderate; (+++), severe gastric intestinal metaplasia.

## Data Availability

The datasets generated and/or analyzed during the current study are not publicly available due to institutional regulations and patient confidentiality, but are available from the corresponding author on reasonable request.

## Abbreviations

GIM, Gastric Intestinal Metaplasia; HP, *Helicobacter pylori*; IBS, Irritable Bowel Syndrome; SIBO, Small Intestinal Bacterial Overgrowth; PXR, Pregnane X Receptor; NF-κB, Nuclear Factor Kappa B.
